# The Only Way Is Up: Active Knee Exoskeleton Reduces Muscular Effort in Quadriceps During Weighted Stair Ascent

**DOI:** 10.1109/lra.2025.3551543

**Published:** 2025-03-14

**Authors:** Vincent S. Boon, Brendon Ortolano, Andrew J. Gunnell, Margaret Meagher, Rosemarie C. Murray, Lukas Gabert, Tommaso Lenzi

**Affiliations:** Department of Biomechanical Engineering, University of Twente, 7522 NB Enschede, The Netherlands; Utah Robotics Center and the Department of Mechanical Engineering, University of Utah, Salt Lake City, UT 84112 USA; Utah Robotics Center and the Department of Mechanical Engineering, University of Utah, Salt Lake City, UT 84112 USA, and also with the Rocky Mountain Center for Occupational and Environmental Health, Salt Lake City, UT 84111 USA; Utah Robotics Center and the Department of Mechanical Engineering, University of Utah, Salt Lake City, UT 84112 USA, and also with the Rocky Mountain Center for Occupational and Environmental Health, Salt Lake City, UT 84111 USA; Utah Robotics Center and the Department of Mechanical Engineering, University of Utah, Salt Lake City, UT 84112 USA; Utah Robotics Center and the Department of Mechanical Engineering, University of Utah, Salt Lake City, UT 84112 USA, and also with the Rocky Mountain Center for Occupational and Environmental Health, Salt Lake City, UT 84111 USA; Utah Robotics Center and the Department of Mechanical Engineering, University of Utah, Salt Lake City, UT 84112 USA, also with the Rocky Mountain Center for Occupational and Environmental Health, Salt Lake City, UT 84111 USA, and also with the Department of Biomedical Engineering, University of Utah, Salt Lake City, UT 84112 USA

**Keywords:** Wearable robotics, physically assistive devices, prosthetics and exoskeletons, EMG, stairs

## Abstract

Firefighters consistently rank stair ascent with gear, which can weigh over 35 kg, as their most demanding activity. Weighted stair climbing requires dynamic motions and large knee torques, which can cause exhaustion in the short term, and overuse injuries in the long term. An active knee exoskeleton could potentially alleviate the burden on the wearer by injecting positive energy at key phases of the gait cycle. Similar devices have reduced the metabolic cost for various locomotion activities in previous studies. However, no information is available on the effect of active knee exoskeletons on muscular effort during prolonged weighted stair ascent. Here we show that our knee exoskeletons reduce the net muscular effort in the lower limbs when ascending several flights of stairs while wearing additional weight. In a task analogous to part of the physical fitness test for firefighters in the US, eight participants climbed stairs for three minutes at a constant pace while wearing a 9.1 kg vest. We compared lower limb muscle activation required to perform the task with and without two bilaterally worn Utah Knee Exoskeletons. We found that bilateral knee assistance reduced average peak quadriceps muscle activation measured through surface electromyography by 32% while reducing overall muscle activity at the quadriceps by 29%. These results suggest that an active knee exoskeleton can lower the overall muscular effort required to ascend stairs while weighted. In turn, this could aid firefighters by preserving energy for fighting fires and reducing overexertion injuries.

## I. Introduction

FIREFIGHTERS rank climbing stairs with their 35-kg equipment as the most physically challenging part of their job [1], [2]. This result is not surprising, considering that metabolic energy expenditure increases by almost 50% when climbing stairs with the additional weight of typical firefighter gear [3]. Moreover, the average firefighter is 39 years old and has an aerobic limit close to the acceptable minimum for their profession [3], [4], [5]. Premature exhaustion can prevent firefighters from performing their job at a desired pace [6]. Moreover, over half of all reported non-fatal firefighting injuries are related to overexertion, causing muscle-tendon strain and ligament sprains [7]. Therefore, reducing the effort of weighted stair climbing for firefighters could benefit task completion rates and long-term health.

The knee joint contributes substantial positive net energy during stair ascent. Moreover, the peak knee torque during stair ascent is more than double that of level ground walking and increases proportionally to the weight added to the trunk [8], [9]. Therefore, we hypothesize that assisting the knee with a powered exoskeleton will reduce effort during weighted stair ascent. Exoskeletons can assist their wearer by augmenting their joint power output [10]. Passive exoskeletons provide assistance by storing and releasing energy the wearer generates during movements. Thus, they can offload the wearer’s joint during moments of peak torque demand, but they have a net-zero energy contribution to the movement. In contrast, active exoskeletons can inject net positive energy into the human-robotic system using their embedded actuators [11]. Previous studies have shown reductions in metabolic cost and muscular effort utilizing both passive and active exoskeletons in various locomotion activities [12], [13], [14], including stair ascent [15]. However, to the best of our knowledge, no study has assessed the effect of knee exoskeleton during weighted stair ascent beyond the analysis of a few strides [16], [17], [18] or only a subsection of the gait cycle [19]. Therefore, although some of these studies indicate that knee exoskeletons can decrease muscular effort at the quadriceps, we are unaware of any conclusive research on the effects of active knee exoskeletons during weighted stair ascent.

In this study, we investigated the effect of a bilateral active knee exoskeleton on muscle effort during prolonged weighted stair ascent. Eight participants climbed stairs for 3 minutes with a 9.1 kg weighted vest (similar in weight to a firefighter’s hose bundle) with and without the exoskeletons. To assess the effect of the exoskeleton on the user’s effort, we collected kinematic and electromyographic (EMG) data from both lower limbs. The results of this study offer insights into the potential benefit of active knee exoskeletons during weighted stair ascent.

## II. Methods

### A. Powered Knee Exoskeleton

**Fig. 1.**
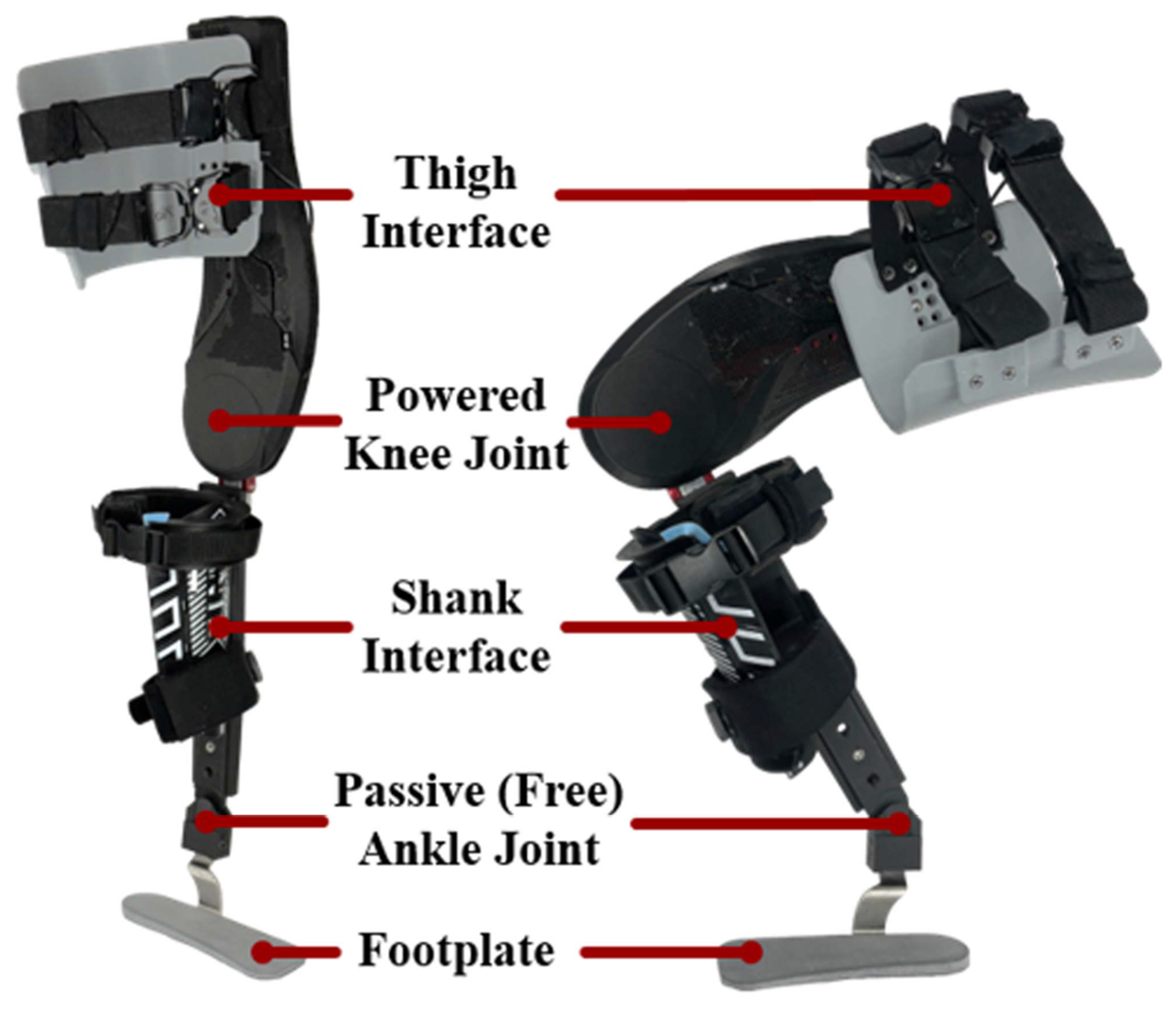
A picture of the Utah Knee Exoskeleton, including covers and physical interfaces. A total of eight physically adjustable settings are present to improve fitting for a wider range of wearers.

In this study, we use two prototypes of the Utah Knee Exoskeleton, shown in Fig. 1. The Utah Knee Exoskeleton consists of an actuated knee joint and a passive ankle joint. The ankle joint uses a free hinge to support the weight of the device without applying torque at the ankle. The knee joint can apply torques of up to 55 Nm, despite the device’s low weight (2.6 kg including battery and physical interfaces). To achieve this high torque density (~21 Nm/kg), the Utah Knee Exoskeleton uses a geared brushless DC motor (EC-22 120 W and 3.8:1 gearbox, Maxon Motors, Switzerland) in combination with a torque-sensitive actuator, which consists of a five-bar linkage with an elastic element similar to [20]. This torque-sensitive actuation (TSA) changes the transmission ratio passively and continuously as a function of the output torque, allowing for a wider range of torque-velocity profiles than a fixed transmission ratio.

The DC motor is powered by a current driver (Everest Core, Ingenia, Spain), which uses an absolute encoder (RM08, RLS, Slovenia) for commutation. Absolute encoders also measure the deformation of the elastic element in the torque-sensitive transmission (iC-MU & MU2S, iC-Haus, Germany) and the angular position of the main output joint (iC-MU & MU18S, iC-Haus, Germany). An inertial measurement unit (IMU, MTi-1, Xsens Technologies B.V., The Netherlands) is attached to the wearer’s thigh to measure its global orientation and rotational velocity in the sagittal plane. The peripherals are interfaced through two 32-bit microcontrollers (PIC32, Microchip Technology, USA). Finally, an embedded computer (Raspberry Pi 4 Compute Module) communicates with the microcontrollers and to a remote computer over Wi-Fi for online data telemetry and control parameter tuning.

A set of semi-rigid interfaces transfers the assistive loads generated by the active knee exoskeleton to the user’s lower limbs. The interfaces comprise a thigh cuff, a shank cuff, and a footplate (Fig. 1). A free hinge connects the shank cuff to a three-quarter length footplate, to preserve natural motion at the ankle and toe joints. The ankle joint and footplate prevent the device from sliding down or twisting about the leg, which ensures that the torque at the knee is accurately applied. During fitting, the interfaces are adjusted to each person to minimize misalignment between the user’s biological knee joint and the powered knee joint axis.

### B. Assistive Controller

The assistance provided by the active knee exoskeleton is determined by the controller represented in Fig. 3, which has feedforward compensations as well as a state-dependant assistive torque profile.

The feedforward compensation torque

(1) $$\tau_{c}=-\left(I{\ddot{\theta }}_{m}+b_{TSA}{\dot{\theta }}_{TSA}+\tau_{fric}\right)$$

lowers the apparent impedance of the exoskeleton to the wearer. ${\dot{\theta }}_{TSA}(rad/s)$ is the velocity of the elastic element’s joint whereas ${\dot{\theta }}_{m}$, ${\ddot{\theta }}_{m}\left(rad/s,rad/s^{2}\right)$ are the motor velocity and acceleration, used in conjunction with the feedforward inertia $I\left({kgm}^{2}\right)$ and the TSA- and motor damping terms $b_{TSA}$, $b_{m}(Nm/s)$. The TSA damping is set to zero when the elastic element flexes, and set to a negative value when it extends, which dampens the release of energy stored in the elastic element. The friction compensation, $\tau_{fric}$, is conditional based on a speed threshold, ${\dot{\theta }}_{m}^{THS}(rad/s)$, and described through

(2) $$\tau_{fric}=\left\{\begin{array}{c} \tau_{coul}\;\text{if}\;\left|{\dot{\theta }}_{m}\right|>{\dot{\theta }}_{m}^{THS},\\ k_{fric}{\dot{\theta }}_{m}\;\text{otherwise}. \end{array}\right.$$

For the assistive torque profile, a state machine splits the wearer’s gait into a flexion and extension phase, depending on the thigh angle relative to the vertical $\left(\theta_{t}\right)$ and the thigh angular velocity $\left({\dot{\theta }}_{t}\right)$, which are illustrated in Fig. 3. The extension phase broadly overlaps with stance phase of the gait cycle, but may begin slightly before foot contact occurs. The transition conditions were based on nominal biomechanics data [21] and refined during pilot testing. While these values worked well for all subjects on our specific step mill, it may need to be adapted for different stair heights. The exoskeleton only applies assistive torque during the extension phase, as nominal biomechanics data of stair ascent indicates that minimal knee torque occurs during the flexion phase [21]. However, assistive torque is not necessarily applied at the transition to the extension phase.

A position-based, trapezoidal torque profile determines the desired assistive joint torque $\tau_{d}$ as a function of thigh angle, $\theta_{t}$. The trapezoidal profile is described with five parameters: The start and end angles of the profile $\left(\theta_{r,s},\theta_{r,e}\right)$ the start and end angle of the profile’s plateau $\left(\theta_{p,s},\theta_{p,e}\right)$, and the peak torque $\tau_{p}$ (Fig. 3). We tuned these five parameters to each subject during the training sessions. The torque assistance profile begins at $\theta_{r,s}$, the thigh angle at which the weight acceptance phase of the stride begins. Setting $\theta_{r,s}$ too high may cause gait instability by applying the torque before weight transfers to the stance leg, or even before foot contact occurs. Too low a starting angle causes greater quadriceps activation, yielding a lost opportunity for effective support. Similarly, $\theta_{p,s}$ is set such that the assistive torque ramps up sufficiently fast to support the increased weight on the stance leg without jerking said leg. Afterwards, the end of peak torque, $\theta_{p,e}$, and the end of support, $\theta_{r,e}$, are set such that the wearer is eased into full stance without overextending their knee. Finally, the peak torque is gradually increased during the training session until the wearer notes that the change in torque feels undesirable. The additional weight carried by the participant during the experiment is assumed to proportionally affect the required (assistive) knee torque.

To realize the total desired torque $\tau =\tau_{c}+\tau_{d}$, a kinematic model of the drive train is combined with a dynamic model of the motor to compute the desired current *I_d_*. This desired current is sent to the motor driver’s internal closed loop current control. We calculate the instantaneous transmission ratio of the drive train from the encoder data on both the motor and the elastic element [20]. The open-loop torque control method described above yields a steady state error of 1.2% in devices with a similar drive train design [20]. The low-level microcontrollers perform these calculations at a rate of 2 kHz. The controller parameters can be set via the embedded computer’s high-level control loop, which runs at 500 Hz. The embedded computer connects to a laptop via WiFi, so that these parameters can be tuned while the exoskeleton is running.

### C. Participant Information

A convenience sample of eight participants with no mobility impairments participated in this study, as described in Table I. The Institutional Review Board at the University of Utah approved the study protocol (IRB_00120712). The subjects provided informed consent to participate in the study, as well as permission to publish photos and videos from the experiment.

### D. Experimental Protocol

**Fig. 2:**
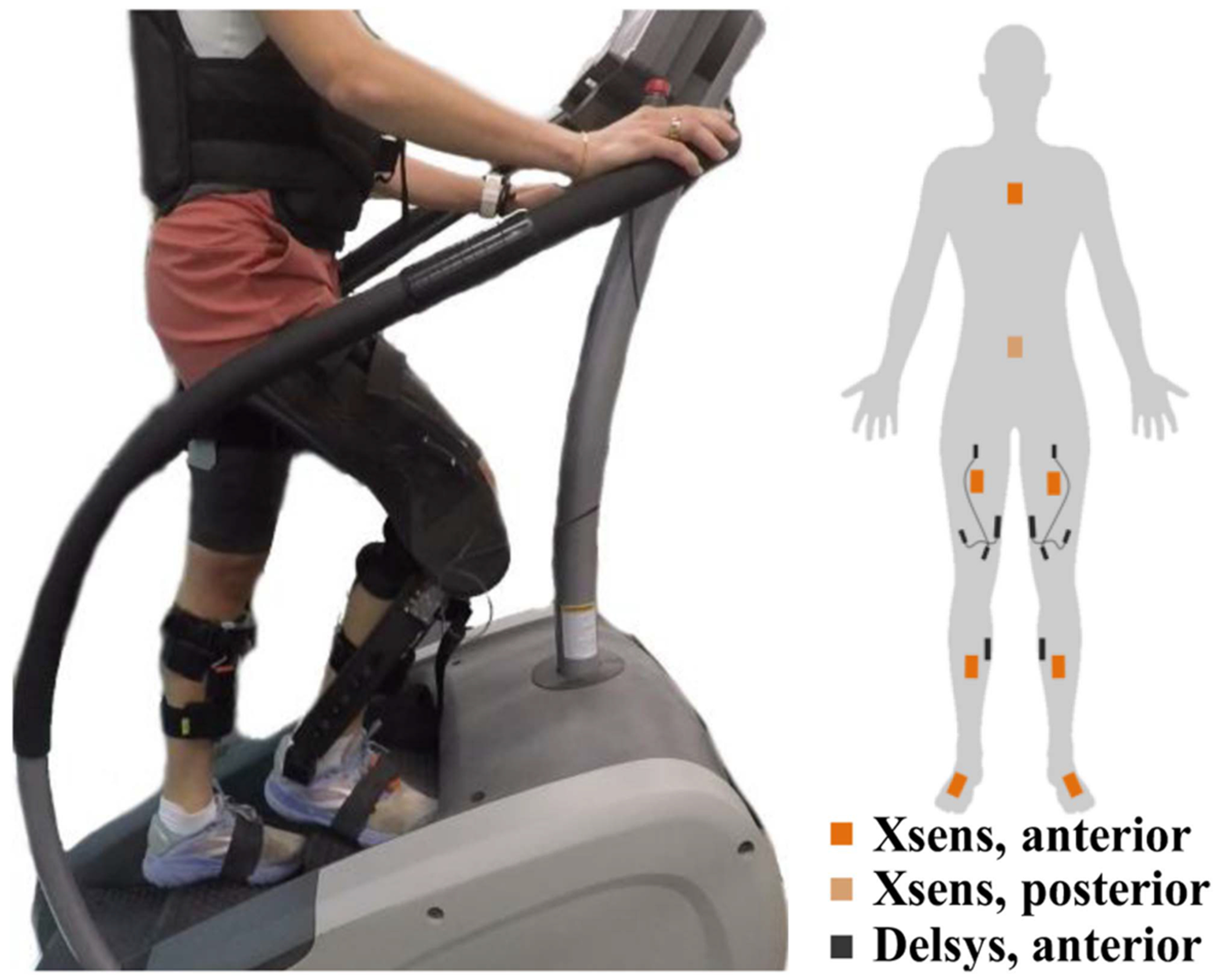
Left, a participant on the step mill with the active knee exoskeletons worn bilaterally while walking up the stairs with a 9.1 kg weighted vest. Right, the placement of Xsens (kinematic) sensors and Delsys (surface electromyography) to gather biomechanical data.

**Fig. 3.**
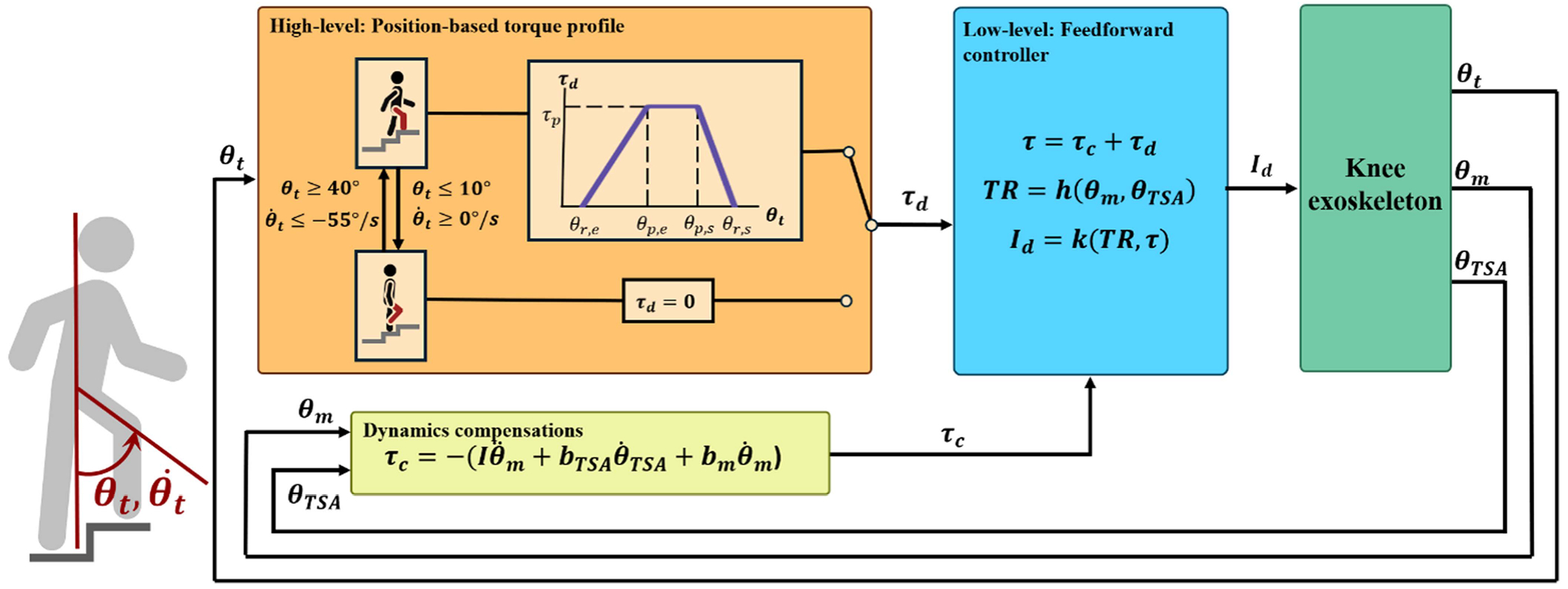
Block diagram of the stair ascent controller. A finite state machine determines if the wearer is in flexion or extension. During extension, a position-based torque profile is employed, while no assistance is given in flexion. Additional impedance compensation torque is provided based on the motion of the motor and passive variable transmission. The (desired) feed-forward current is computed using the instantaneous transmission ratio of the drive train.

Prior to data collection, participants familiarized themselves with the device during two 45-minute training sessions. During these sessions, the exoskeleton interfaces were adjusted to the user. After familiarization, the participants donned electromyography and motion capture sensors, shown in Fig. 2. We measured surface electromyography (sEMG), a proxy for muscular effort [22]. We placed four wireless sEMG sensors on each leg at the rectus femoris (RF), vastus medialis (VM), vastus lateralis (VL) (Trigno Quattro Sensor, Delsys Inc., USA), and gastrocnemius medialis (GM) (Trigno Avanti Sensor, Delsys Inc., USA). The ground units of the Trigno Quattro sensor also house an inertial measurement unit (IMU), which we use for data segmentation in post-processing.

We prepared the skin and placed the sensors according to SENIAM guidelines [23], with the exception of the gastrocnemius medialis sensor, which is located slightly more proximally to eliminate contact with the exoskeleton interface and reduce the potential for motion artifacts. We wrapped the sensors on the upper leg to minimize motion artifacts from interface deformation. The sEMG data is acquired through EMGWorks Analysis v4.8.0 (Delsys Inc., USA).

To measure joint kinematics, participants wore eight wireless inertial motion capture sensors on their lower limbs, pelvis, and sternum (Xsens MVN Awinda, Xsens Technologies B.V., Enschede, The Netherlands), drawn in orange in Fig. 2. A separate computer records the kinematic joint data through MVN Analyze 2020.0 (Xsens Technologies B.V., Enschede, The Netherlands). After sensor placement, subjects performed a calibration sequence. A dedicated trigger panel from DelSys synchronized the data acquisition between sEMG and motion capture systems [24]. The Utah Knee Exoskeleton recorded sensor data separately on its embedded computers.

Participants completed a task similar to event one of the Candidate Physical Abilities Test (CPAT) for firefighters in the United States [25], which requires candidates to ascend a step mill at a speed of 60 steps/min for three minutes, while wearing 34 kg of added weight and without touching the handrails. In our study, participants similarly ascended a step mill (StepMill 3, StairMaster, United States of America) at 60 steps/min for three minutes without using the handrails for weight bearing. The steps were 15 cm tall and 23 cm deep. However, because firefighter candidates are recommended to complete several weeks of fitness training before attempting the CPAT [25], and our participants were drawn from the general population with no prior training, we asked participants to wear 9.1 kg weighted vest rather than the full 34 kg. The participants completed the test twice: once with the exoskeleton providing assistive torque and once without the exoskeleton. Each trial began with a ninety-second warmup while the step mill slowly accelerated, followed by a three-minute data collection session. In the exoskeleton condition, the assistive peak torque slowly increased throughout the warm-up period, in order to ease the wearer into the assistance and avoid excessive co-contractions [26], [27]. In between trials, the participants rested for at least thirty minutes. The motion capture sensors were replaced and recalibrated between trials to accommodate donning or doffing the exoskeleton interfaces. Half the participants completed the exoskeleton trial first, while the other half started without the exoskeleton in an effort to average the effects of fatigue between conditions.

### E. Post-Processing

The data was analysed in Matlab R2022b (MathWorks, USA). sEMG data are exported using EMGWorks Analyze (Delsys Inc., USA), whereas the joint trajectories are exported through Xsens’ Matlab library [28]. Biological joint kinematics are high-pass filtered at 0.15 Hz using a second-order Butterworth filter. The sEMG data are band-pass filtered (20-450 Hz, second order Butterworth filter), rectified, and low-pass filtered using a 5 Hz second-order Butterworth filter. We compute peak activation and area under the curve for each stride, with the latter considered a proxy for total muscular effort. The calculated exoskeleton output torque is low-pass filtered with a 100 Hz second-order Butterworth filter, while output joint velocity data is low-pass filtered with a 40 Hz second-order Butterworth filter. Exoskeleton computed output torque and power are bodyweight normalized. All filter orders and frequencies are based on visual inspection of the raw and filtered data in both the temporal- and frequency domain.

After filtering, we segmented the data by stride. As foot contact data was not available, we segmented at the peak thigh angle relative to the vertical, which likely precedes heel strike slightly [21]. We then interpolated each stride to 1000 samples. Next, we normalized the filtered sEMG data with respect to the mean peak activation of the respective muscle in the no-exoskeleton condition. Finally, we calculated key outcome measures, such as minimum hip flexion angle, peak knee extension speed, and mean trunk lean.

We tested key metrics of the kinematics and sEMG data for statistically significant differences between the exoskeleton and no-exoskeleton conditions using paired t-tests. While all participants completed the same task, differences in height or body mass could make the task relatively more or less challenging. In addition, we tuned the exoskeleton individually for each subject. To assess the impact of these individual differences on performance, we used Pearson’s correlation coefficient to test for correlations between participant height and mass, and the peak level of assistive torque. We used Pearson’s correlation coefficient to test for correlations between the peak torque and power and changes in peak and integral sEMG activation and knee extension speed. The alpha level for all statistical tests is set to *α* = 0.05. When applicable, we applied the Bonferroni correction for multiple comparisons.

## II. Results

### A. Exoskeleton Kinetics

**Fig. 4.**
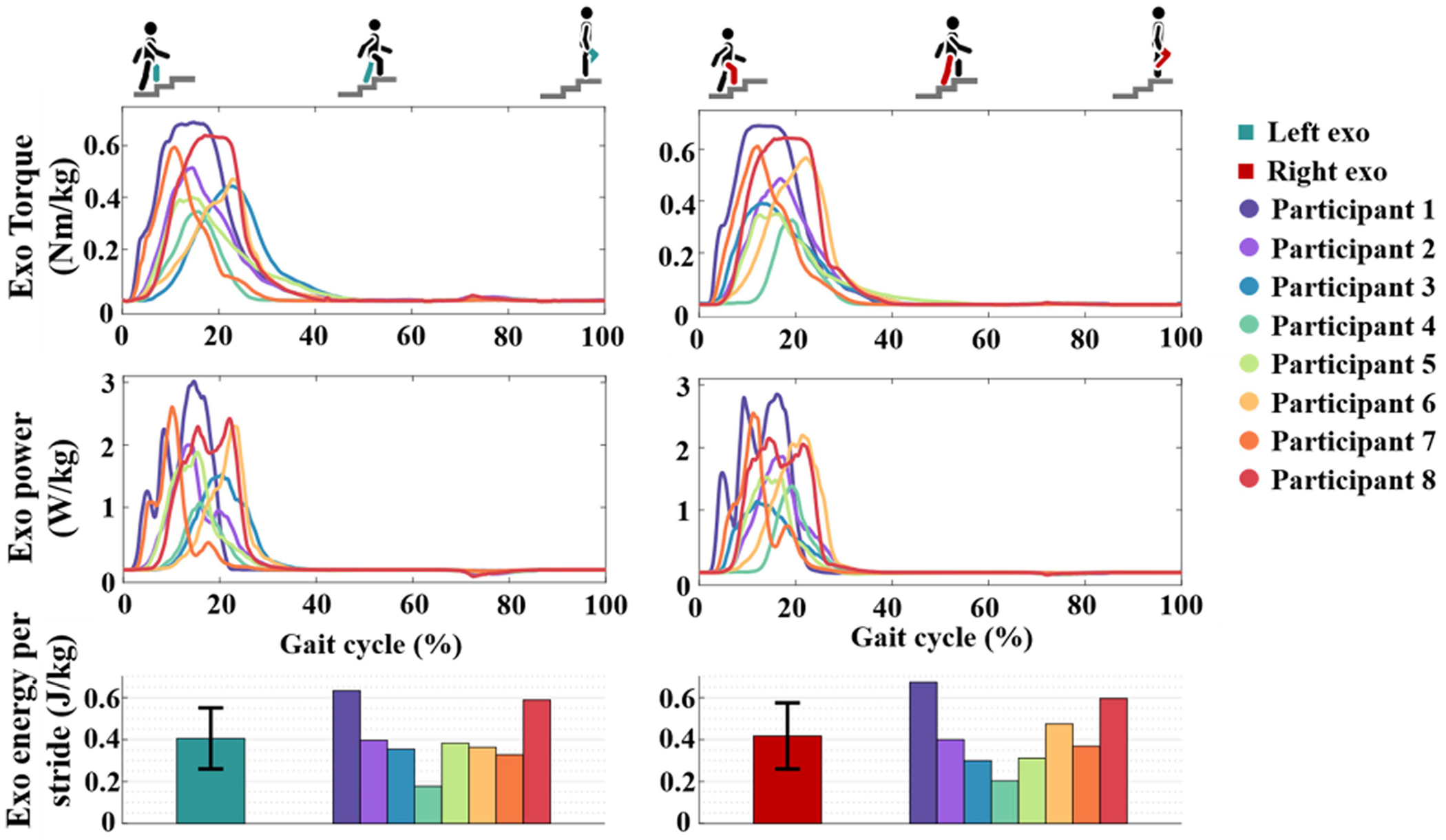
Mean bodyweight normalized exoskeleton torque (top), power (middle) and energy generated per stride (bottom) on the left (left column) and right (right column) side for each participant. Extension is defined as positive. The gait cycle is segmented based on the maximum angle of the thigh per stride.

Fig. 4 displays the output torque and power trajectories of both exoskeletons across the gait cycle for each participant. The trapezoidal position-based torque profile manifests as a smoother and more bell curve-like profile in the time domain. The mean peak assistive torque ranged from 0.38 Nm/kg (participant 4) to 0.71 Nm/kg (participant 1) based on tuning, which corresponds to 42% – 79% of the nominal peak biological torque for unweighted stair ascent [21]. On average, the torque profiles (including compensations) start around 4% of the gait cycle and end at 40%.

The mean peak bodyweight normalized power ranged from 1.2 W/kg (participant 4) to 2.9 W/kg (participant 1), which is 56% and 133% of the peak biological knee power during unweighted stair ascent, respectively [21]. As described in Section II, the controller only provided positive power during the stance phase, with negligible power generated during swing. Exoskeleton power oscillated somewhat during peak assistance, particularly for participants with higher torques.

Finally, the bottom row of Fig. 4 indicates that both exoskeletons generated an average bodyweight normalized energy of approximately 0.4 J/kg over a stride.

### B. Joint Kinematics

**Fig. 5.**
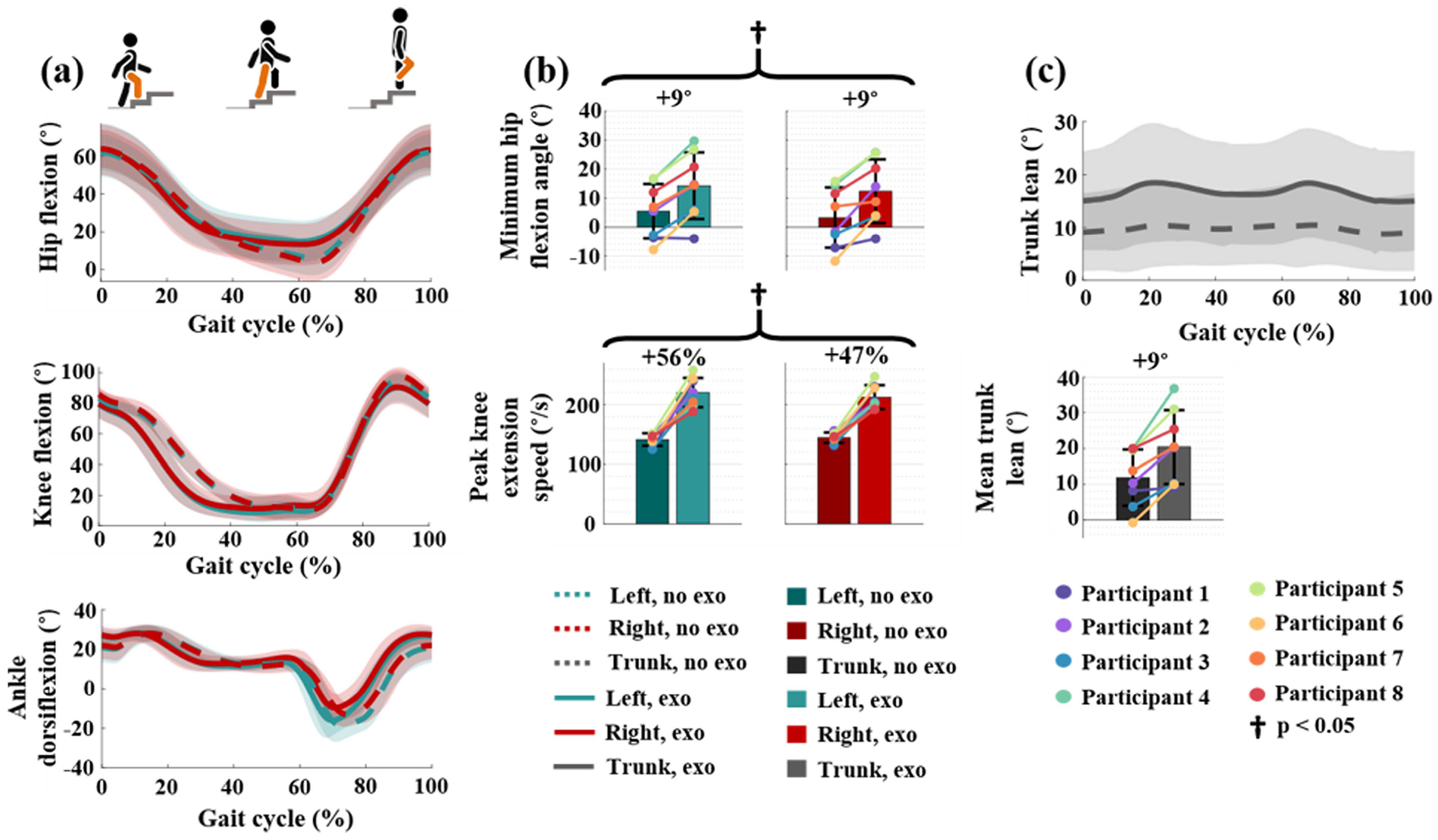
(a) The mean kinematics of the lower limb joints plotted over the gait cycle. (b) The minimum hip flexion angle (above) and peak knee extension speed (below). (c) The mean trunk kinematics plotted over the gait cycle (above) and the mean trunk angle (below). Trunk kinematics are segmented based on the strides of the left leg. In line plots, the colored shading indicates a range of one standard deviation above and below the mean. In bar plots, colored dots represent individual subjects. Crosses above the bar plots indicate statistically significant differences between the two testing conditions (p < 0.05).

The joint kinematics are presented in Fig. 5. Column (a) plots the joint kinematics averaged over all participants for each condition. At the knee level, Fig. 5 shows an increased knee extension velocity at the start of the gait cycle. Each participant increased their knee extension speed. On average, the knee extended 52% faster (p = 3.4 × 10^−13^, *n_tests_* = 2), with no significant difference between legs (no exoskeleton condition: p = 0.53, exoskeleton condition: p = 0.50, *n_tests_* = 2). Moreover, the knee extension phase shortens by approximately 10% of the gait cycle in the exoskeleton condition. On the other hand, more time is spent at maximum knee extension before the leg is lifted to the next step.

In the trunk kinematic data, Fig. 5(c) shows that participants lean further forward when wearing the exoskeleton during stair ascent by an average of 9°, although this change is not statistically significant (p = 0.08, *n*_tests_ = 1).

Regarding hip kinematics, the most notable difference is that the minimum hip flexion angle decreased by an average of 9° in the exoskeleton condition, p = 0.02, *n*_tests_ = 2. As with the knee, there is no significant difference between the left and right side for the minimum hip flexion angle (no exoskeleton: p = 0.67, exoskeleton: p = 0.73, *n*_tests_ = 2). Finally, the main variation in ankle kinematics between conditions appears during the swing phase. In the exoskeleton condition, peak plantarflexion is held for a shorter time while peak dorsiflexion is held longer, from the end of swing until weight acceptance.

### C. Muscle Activation

**Fig. 6.**
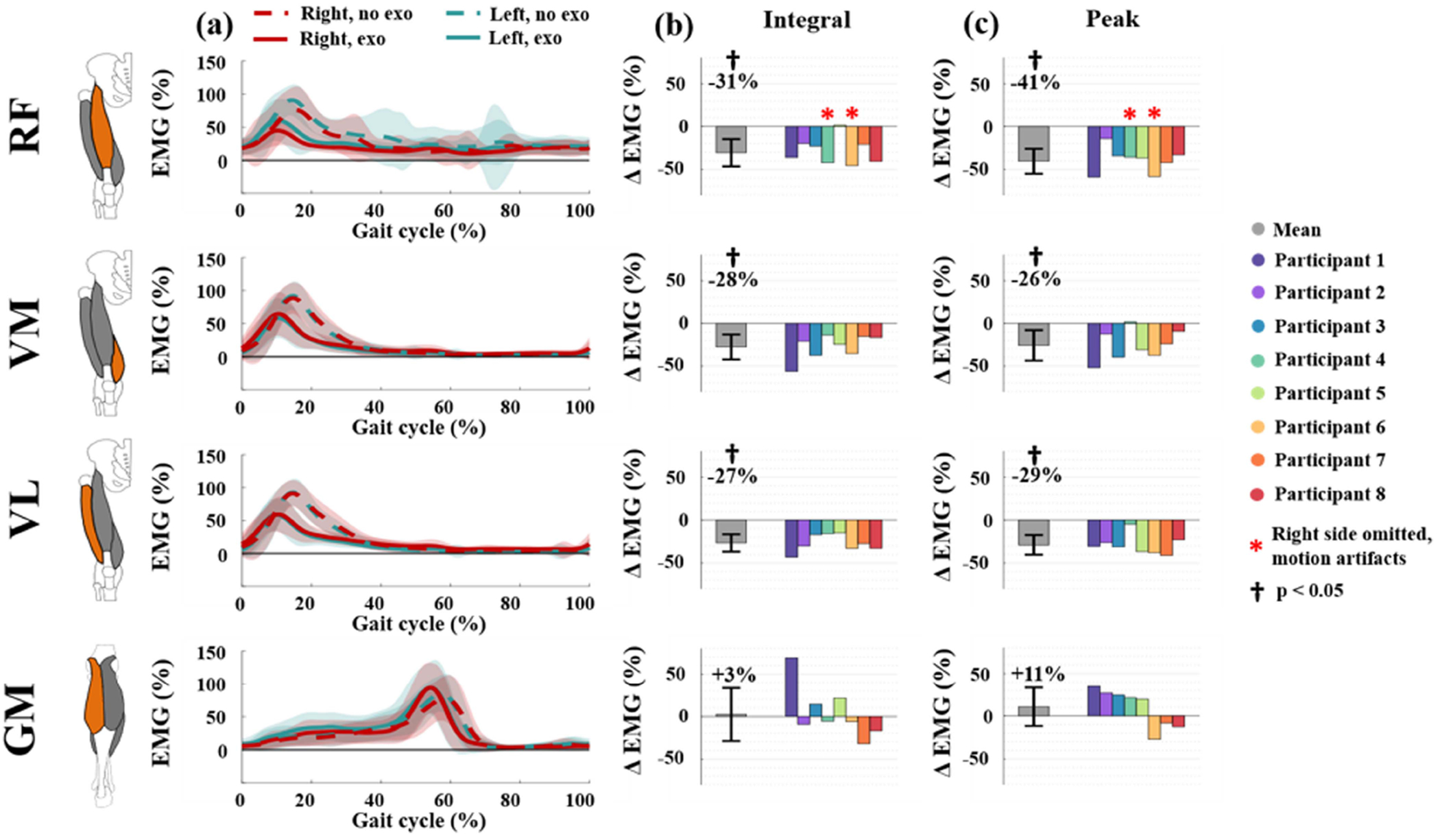
Muscle activation of the rectus femoris (RF), vastus medialis (VM), vastus lateralis (VL), and gastrocnemius medialis (GM). (a) The mean muscle activation across the gait cycle. The colored shading indicates a range of one standard deviation above and below the mean. (b) The change in the mean muscle activation integrated over the stride. (c) The change in the mean peak muscle activation per stride. EMG data is normalized by the mean peak activation during the no-exoskeleton trial. The bar graphs indicate the percentage change between the no-exoskeleton trial and the exoskeleton trial, averaged over both legs. The grey bars represent the average of all participants, with crosses indicating statistical significance (p < 0.05).

The sEMG data are shown in Fig. 6. The right rectus femoris data of participant 4 and participant 6 are excluded due to substantial motion artifacts. In Fig. 6(a), a good agreement can be observed between the mean activation patterns of the left and right muscles in the same condition. Temporally, the mean peak of each activation pattern occurs roughly 5% earlier with the exoskeleton than without.

When wearing the exoskeleton, the participants reduced activation of all three measured quadriceps muscles. The mean integral of the sEMG signal, a proxy for total muscle activation, significantly decreased during the exoskeleton trial compared to the no-exoskeleton trial (RF: −31%, p = 0.0015; VM: −28%, p = 0.0011; VL: −27%, p = 1.410^−4^). Similarly, the peak sEMG value, analogous to the peak muscle activation, reduced significantly for all quadriceps muscles while participants wore the active knee exoskeletons (RF: −41%, p = 1.2 × 10^−4^; VM: −26%, p = 0.0048; VL: −29%, p = 1.9 × 10^−4^).

In contrast, the gastrocnemius muscle may activate more when using the exoskeleton. The mean integral gastrocnemius medialis activation increases slightly when participants wear the active knee exoskeletons, although this change is not significant (GM: +3%, p = 0.71). Similarly, the peak activation of the GM increased by 11% during the exoskeleton trials, although this change was not significant (p = 0.26).

There was a trend toward subjects with greater mass or height receiving larger peak torques, but these correlations were not significant (p = 0.079, p = 0.16; respectively). There were no statistically significant correlations between the peak torque or peak power values and the change in integral sEMG, peak sEMG, or knee extension speed. This suggests that differences in individuals may have been accounted for through changes in the control parameters, or that a larger study is needed to detect these differences.

## IV. Discussion

In this letter, we investigated the effect of bilaterally wearing an active knee exoskeleton on muscular effort during prolonged weighted stair ascent. In this experimental study with eight individuals, we found that the overall muscular effort in the quadriceps decreased by an average of 29% during a three-minute weighted stair ascent trial when assisted by two Utah Knee Exoskeletons. Moreover, the mean peak activation of the quadriceps decreased by 32%. These are the first results known to the researchers that indicate how active knee exoskeletons can provide a net decrease in muscular effort for individuals in an occupational setting. By decreasing the overall muscular effort during weighted stair climbing, we hypothesize that firefighters will have more stamina remaining to fight the fire once on location. Furthermore, decreasing the peak activation in this strenuous activity might also decrease the resulting peak load on the lower-limb muscle-tendon units. This implies that an active knee exoskeleton might not only reduce the task effort, but also minimize overexertion injuries during weighted stair climbing. Finally, as firefighters in the US are near the aerobic metabolic limit needed to perform their duties [3], [4], [5], future studies could investigate the metabolic impact of these devices during weighted stair ascent [29].

Regarding the kinematic results, we observed an increase in maximum knee extension speed by an average of 52% when participants were wearing the active knee exoskeletons. While participants extended the knee more quickly, they paused at maximum knee extension before beginning the weight transfer for the following step. Since the step mill constrained the participants’ speed, we hypothesize that this pause would disappear when climbing stairs freely, with the overall speed increasing instead. If this were the case, the active knee exoskeletons would not only reduce the muscular effort required to complete a weighted stair climbing task, but also increase the task completion speed. However, future studies must test this hypothesis using a staircase instead of a step mill.

More closely observing the joint kinematics, the decrease in maximum hip extension angle seems closely related to the increase in the trunk lean angle of participants. Although the exact reason for this behavior is unknown, we suggest some potential contributing factors. First, individuals had only a brief training period with the exoskeletons, and may have adopted a more cautious stair climbing strategy in response. Participants might lean forward in order to watch their foot placement on the step mill. Second, it could reflect a suboptimal tuning of the *θ_r,s_* parameter. If this value is set too low, then the knee extension torque will push the pelvis backward as well as upward. The subject would then need to lean the trunk forward to keep their mass centered over the step. Third, it could be a general response to an increased total torque at the knee. The increase in knee extension speed when wearing the exoskeleton suggests that despite the reduction in quadriceps activation, the sum of the biological and exoskeleton torque in the exoskeleton condition is greater than the biological torque in the no exoskeleton condition. In this case, given more time to adapt to the device, subjects may be able to further reduce muscle activation and return to typical stair climbing kinematics. In the future, longer training and tuning times might prevent these deviations in kinematics and minimize the impact of active knee exoskeletons on the rest of the wearer’s body.

In both kinematics (Fig. 5) and muscular activation patterns (Fig. 6), subjects experienced notably symmetric outcomes. Intriguingly, this occurs despite differences in the timing and amplitude of the assistive profiles, shown in Fig. 4. This discrepancy might have various causes. Each exoskeleton was tuned individually, prioritizing participants’ perceived symmetry of support over identical control parameters. However, the two exoskeletons may have slightly different dynamic behavior due to differences in fabrication. Small differences in the interface fit between the two legs could affect the torque transfer to the body. Moreover, a slight difference in the placement or orientation of the IMUs used to determine the thigh angle might impact the support profile of the exoskeleton during stair ascent. Therefore, we advise those studying bilaterally worn assistive devices to tune for symmetry of output metrics rather than matching control parameters.

This study was designed to answer the specific question of whether active knee assistance could reduce muscle effort during weighted stair ascent. To do so, we employed a state-dependent, position-based assistive torque profile because its simplicity allowed for intuitive implementation and tuning. However, the controller may need substantial adaptation to translate these results to the real world.

There are some biomechanical differences between step mill ascent and overground stair ascent, such as a shorter duration stance phase, which may affect the control strategy [30]. In addition, the controller should accommodate changes in speed, added weight, and stair height. The position-based nature of our current controller gives the user control over the speed of the movement, although the amount of torque provided would not change. Similarly, the current controller is robust to changes in the mass of the participant, but the torque provided does not change in response. Finally, we expect that the thigh position thresholds of the controller may need to be adjusted to accommodate different stair heights.

Determining the optimal knee exoskeleton control strategy for weighted stair ascent in the real world will require further study. However, control strategies developed for other devices can offer insight into the problem. Hood et al. developed a powered knee prosthesis controller that modulates extension torque based on knee angle at the start of stance, which allows it to apply larger torques for larger stair heights [31]. Cowan et al extended this work to design a controller that transitions between stair ascent and walking without classification [32]. Cortino et al. used a phase variable to determine desired joint angles in a stair ascent controller for a powered prosthesis [33]. Lim et al developed a delayed output feedback controller for a hip exoskeleton to assist users in both level ground walking and stair ascent [34]. Molinaro et al developed a hip exoskeleton controller that uses a temporal convolutional network to estimate the user’s hip flexion/extension moments, which adjusts the torque output to accommodate a range of stair heights [35]. Creveling et al. developed an EMG-based controller for a powered knee prosthesis that enables users to climb stairs at varying speeds and amounts of added weight [36]. Adapting these control strategies or developing new ones could enable powered knee exoskeletons to effectively assist users in occupational settings.

In conclusion, this study shows that active knee exoskeletons can reduce the overall- and peak muscular effort of eight individuals performing weighted stair ascent for several minutes. In the future, these assistive devices could potentially reduce the muscular loads on firefighters, while increasing stair climbing speed. This study provides a first step in understanding the effect of active knee assistance on strenuous occupational activities such as weighted stair ascent.

## Figures and Tables

**TABLE I:** Subject Information

Participant	1	2	3	4	5	6	7	8
**Height (cm)**	191	170	185	163	170	165	173	165
**Weight (kg)**	72	60	75	54	61	66	73	54
**Age**	22	21	21	26	20	29	23	24
**Gender**	M	F	M	F	F	M	M	F
**Peak Torque (N)**	51	35	34	21	26	39	50	37
